# Transcultural adaptation and validation of the Serbian version of the colorectal-specific quality of life questionnaire FACT-C

**DOI:** 10.1371/journal.pone.0263110

**Published:** 2022-02-03

**Authors:** Jelena Ilic-Zivojinovic, Igor Krdzic, Ana Jovanovic, Danka Vukasinovic, Branislav Ilic, Aleksandar Gavrilovic, Ivan Soldatovic

**Affiliations:** 1 Faculty of Medicine, Institute of Hygiene and Medical Ecology, University of Belgrade, Belgrade, Serbia; 2 Department of Colorectal Surgery, University Clinical Hospital Center Zvezdara, Surgery Clinic “Nikola Spasić”, Belgrade, Serbia; 3 School of Dental Medicine, Clinic for Oral Surgery, University of Belgrade, Belgrade, Serbia; 4 Department of Neurology, Faculty of Medical Sciences, University of Kragujevac, Kragujevac, Serbia; 5 Clinic of Neurology, University Clinical Center Kragujevac, Kragujevac, Serbia; 6 Faculty of Medicine, Institute of Medical Statistics and Informatic, University of Belgrade, Belgrade, Serbia; Tabriz University of Medical Sciences, ISLAMIC REPUBLIC OF IRAN

## Abstract

**Objective:**

Transcultural adaptation and validation of the FACT-C questionnaire to Serbian language.

**Methods:**

The study included 131 patients with colorectal cancer. Translation included standard forward and backward translation from original language to Serbian and back. Pilot testing of the questionnaire was conducted on 10 patients with diagnosed colorectal cancer. The questionnaires EORTC-QLQ-C30 and DASS will be used as validated tools to evaluate validity of examined, FACT-C questionnaire.

**Results:**

The FACT-C demonstrated satisfactory construct validity using Cronbach’s alpha. Satisfactory concurrent validity was demonstrated using correlations with EORTC-QLQ-C30 and DASS questionnaires. High reproducibility was demonstrated using repeated questionnaires on 30 patients two weeks after the first interview.

**Conclusion:**

The Serbian version of the FACT-C was demonstrated to have satisfactory applicability, reliability and validity in Serbian patients with colorectal cancer. It can be considered as a valid colorectal cancer specific health related quality of life tool for the Serbian population.

## Introduction

Colorectal cancer (CRC) represents the second leading cause of cancer deaths worldwide [1]. The highest incidence rates are in Australia and New Zealand, Europe, and North America, and the lowest rates are found in Africa and South-Central Asia [1]. It is the third leading cause of cancer death among adults younger than age 50, second among men and the fourth among women in the USA [2, 3].

In the Republic of Serbia, colorectal cancer (CRC) is the second most frequent cancer in both sexes, with 3800 new cases and 2300 deaths annually [4]. According to the data of the Register for Cancer of the Institute for Public Health of Serbia “Dr Milan Jovanović Batut” for the year 2012 CRC was the second most frequent cancer in males for both incidence and mortality and the second leading cause of mortality due to malignancy, for females [5].

CRC has multiple impacts on health-related quality of life (HRQoL) caused by its symptoms and the consequence of the treatment. It may result in psychophysical or functional impairment or disruption of social and family interactions or can cause a long-lasting depressive mood which can influence on low HRQoL [6].

QoL is a multidimensional, subjective and patient-reported measure for evaluating the full impact of the disease on the individuals, their family and their community [6].

There are two HRQoL measures specific for CRC patients. One is The European Organization for Research and Treatment of Cancer (EORTC), colorectal module QLQ-CR38 and the other is North American Functional Assessment of Cancer Therapy (FACIT) questionnaire FACT-C, version 4, which combine FACT-G (general) with a CRC subscale (CCS) from the Center on Outcomes, Research, and Education (CORE) [7–9]. This self-report instrument combines specific concerns related to colorectal cancer with concerns that are common to all cancer patients as assessed with the FACT-General (FACT-G).

The aim of this study was to test psychometric properties of the Serbian FACT-C (version 4) and to determine whether it could be a valid instrument tool for colorectal cancer patients’ quality of life in Serbian population.

## Materials and methods

### Questionnaires

European Organization for Research and Treatment of Cancer (EORTC) developed a generic questionnaire—the EORTC Quality of Life Questionnaire-Core 30-questions (QLQ-C30) for QOL assessments in all patients with Cancer. The EORTC-QLQ-C30 has been one of the most widely used cancer questionnaires and has been translated and linguistically validated into more than 60 languages and Serbian among them [10]. The EORTC QLQ-C30 incorporates nine multi-item scales: five functional scales (Physical, Role, Cognitive, Emotional and Social Functioning); three symptom scales (Fatigue, Pain and Nausea/Vomiting); and a Global Health Status/QoL scale. Six single item scales are also included (Dyspnea, Insomnia, Appetite Loss, Constipation, Diarrhea and Financial Difficulties) [11]. The colorectal module QLQ-CR38 is a supplementary questionnaire for assessing specific QoL issues relevant to the patients with colorectal cancer [12]. This module is not validated in Serbian, so in our investigation we used the EORTC-QLQ-C30.

Depression Anxiety Stress Scales (DASS) questionnaire, validated in Serbia, is an instrument designed to measure the three related negative emotional states of depression, anxiety and stress [13]. The scale, proposed by Australian Psychological Society, was developed by Lovibond in 1995 and has four-point Likert scales, consisting of 42 items [14]. Items refer to the past week; and scores range from 0, “Did not apply to me at all” to 4, “Applied to me very much, or most of the time”.

The questionnaires EORTC-QLQ-C30 and DASS will be used as validated questionnaires for dimension comparisons with examined, FACT-C questionnaire.

The FACT-C is available in more than 30 languages, and validated among Spanish, English, French, Korean, and Chinese patients, permitting cross-cultural comparisons of people from diverse backgrounds [15–19].

This questionnaire is designed for use with patients with cancer with specific concern related to colorectal cancer. Each item scores on a five-point Likert scale (Not at all = 0, A little bit = 1, Some-what = 2, Quite a bit = 3, Very much = 4) and reports the patients QoL during the previous 7 days. The scale items consist of five domains: Physical Well-Being (PWB– 7 items), Social/Family Well-Being (SWB– 7 items), Emotional Well-Being (EWB– 6 items), and Functional Well-Being (FWB– 7 items) and Colorectal Cancer Subscale (CCS– 9 items) with questions related to potential concern to patients about the therapy, but with two last items on ostomy appliance not currently scored because only a small percentage of all CRC patients had a colostomy. Item scores for PWB and EWB are calculated reversely. Domain scores are obtained as the sum of all the individual item scores and they are multiplied by the number of the items, and then divided by the number of the items answered. To derive a FACIT—C total score we summarized the domain scores ranging from 0 (worst QoL) to 136 (best QoL) [20, 21].

### Translation and cultural adaptation

The translation of FACT-C was according to the internationally accepted methodology for cross-cultural adaptation of questionnaires and following standard FACIT translation methodology. Two independent translators, working independently from one another, initially provided a forward translation from English into Serbian. Then, the third translator reviewed the English and the two forward translations. A back translation performed by a fourth translator, from Serbian into English, was reviewed by translation project team. The final step, performed by the first author and a clinical expert team, provided the feedback on the acceptability of the translation and back-translation. All these five versions of the questionnaire were submitted to the license holder who incorporates all of them into the FACT-C format.

### Pre-testing

Pilot testing of the pre-final Serbian test version of the questionnaire was conducted on 10 randomized selected patients with diagnosed CRC, and the distribution of patient age was similar to the 131 samples, average 67.0±8.3 [54–77]. The included patients were had confirmed CRC, they were able to read and write Serbian and could give verbally informed consent. The questionnaires were self-administrated and every patient answered the questions from questionnaire together with Patient Interview form prepared by FACIT organization. Patients were asked to read all questions, even those that do not seem relevant, in order to evaluate the wording of the questions and statements. None of the patients found the questions difficult to understand, offensive, irrelevant, disturbing or upsetting. The data from pilot testing were submitted and approval was obtained from FACT-C questionnaire holder.

### Patients

The study included 131 patients from University Clinic for Surgery “Nikola Spasić” of Clinical Center Zvezdara, in Belgrade, Serbia. All the patients signed a written consent form and the study was approved by the Ethics Committee of the Medical Faculty University of Belgrade (No 2650/X-16). FACIT organization approved the use of the FACT-C questionnaire, and the licenses for the other questionnaires which are not in public domain were obtained by their license holders.

Enrolled patients had confirmed CRC, age between 18 and 65, with life expectancy more than 6 months. They were all Serbian native–speakers, able to communicate, with no cognitive impairment or psychosis. All patients were recruited by random sampling. The patients filled out the questionnaires by themselves and for those who had visual, literacy or some other difficulties we provided a trained interviewer.

### Data analysis

Results are presented as count (%), means ± standard deviation depending on data type. All p values less than 0.05 were considered significant. All data were analyzed using SPSS 20.0 (IBM Corp. Released 2011. IBM SPSS Statistics for Windows, Version 20.0. Armonk, NY: IBM Corp.) and R 3.4.2. (R Core Team (2017). R: A language and environment for statistical computing. R Foundation for Statistical Computing, Vienna, Austria. URL https://www.R-project.org/.).

### Construct validity

Internal consistency or homogeneity—shows if all subparts (dimensions) of an instrument measure the same characteristic through Cronbach’s alpha coefficient, as satisfactory when is close to 0.60 or ideal when is higher than 0.7 [22]. Internal consistency was measured using Cronbach’s alpha coefficient.

Concurrent validity was assessed by correlating the FACT-C scores with the domain and composite scores of other HRQOL instruments. We used Pearson correlation to compare FACT-C with EORTC-QLQ-C30 questionnaire. The FACT-C scores were correlated with DASS scores, as stress, depression and anxiety are well-known confounding factors in the assessment of HRQoL.

### Reliability

Reliability, as the consistency of measurement repetition and the ability to reproduce a consistent result in time and space, or from different observers [22] was assessed by intra-class correlation (ICC). Test-retest reliability was also assessed by pared t-test between differences of the FACT-C subscales and total scores. The interpretation of the result according to Landis and Koch [23] suggests that the substantial agreement of items within the same subscale is 0.61–0.80 or 0.81–1.00 when strength of agreement is almost perfect.

## Results

### Descriptive statistics of examined sample

The characteristics of the sample are listed in Table 1. The majority of patients are the eldest (seventh decade in average). Nearly half of the subjects are males. The majority was married and had secondary high school. Half of the patients had classic surgery and one third had laparoscopic. The minority had complication and received radiotherapy and chemotherapy before and after treatment.

**Table 1:** Socio-demographic characteristics of study subjects.

	N (%) / Mean±SD
Gender male	71 (54,2)
Age (yrs)	68.49±9.89
Marital status	
Married	89 (67.9)
Non-married	7 (5.3)
Divorced	12 (9.2)
Widowed	23 (17.6)
Education level	
Primary	20 (15.3)
Secondary	71 (54.2)
High/university	36 (27.5)
No formal school	4 (3.1)
Body mass index kg/m^2^	25.89±4.30
Stage	
I	17 (30.9)
II	16 (29.1)
III	14 (25.5)
IV	8 (14.5)
Treatment	
Classic surgery	72 (55.0)
Laparoscopic surgery	36 (27.5)
No surgery	23 (17.6)
Radiotherapy and chemotherapy before	3 (2.3)
Radiotherapy and chemotherapy after	23 (17.6)
Complication	12 (9.2)
Hospitalization	31 (23.7)
Duration of hospitalization	
<5 days	19 (14.5)
5–10 days	52 (39.7)
>10 days	60 (45.8)

All mean values, standard deviation, floor and ceiling effects of all subscales of FACT-C are presented in Table 2.

**Table 2:** Descriptive statistics, ceiling and floor effects of health-related quality of life questionnaire scores.

Questionnaire/subscale	Mean±SD	Cronbach’s Alpha	Floor effect (%)	Ceiling effect (%)
FACT-C				
PWB (7 items)	23.54±4.52	0.77	0	16.8
SWB (7 items)	24.37±3.97	0.76	0	21.4
EWB (6 items)	20.04±4.17	0.55	0	16.0
FWB (7 items)	20.66±4.86	0.75	0	3.1
CCS (7 items)	21.60±4.13	0.53	0	1.5
FACT-C TOI (21 items)	65.80±11.34		0	0.8
FACT-G total score (27 items)	88.61±12.65		0	0.8
FACT-C total score (34 items)	110.21±15.46		0	0

FACT-C subscales: PWB—Physical Well-Being, SWB—Social/Family Well-Being, EWB -Emotional Well-Being, FWB—Functional Well-Being, CCS—Colorectal Cancer Subscale, TOI—trial outcome index, FACT-G—Functional Assessment of Cancer Therapy—General, FACT-C—Functional Assessment of Cancer Therapy—Colorectal

### Construct validity

Internal consistency in Table 3 shows Cronbach’s alpha coefficients if the items are deleted. All subscales exceeded the acceptable level for Cronbach’s alpha coefficients. In EWB subscale, when questions are being removed the Cronbach’s alpha significantly increases to 0.78, when question “I am satisfied with how I am coping with my illness” is being removed. Similar problem is with CCS subscale. In this subscale, several questions have opposite direction of scoring and the total scale reliability is low, below 0.60.

**Table 3:** Mean values, standard deviation and corrected Cronbach’s alpha coefficients for all the subscales of the Serbian FACT-C questionnaire.

Item	PWB		SWB		EWB		FWB		CCS	
	mean±sd	α* (del)	mean±sd	α* (del)	mean±sd	α* (del)	mean±sd	α* (del)	mean±sd	α* (del)
1	1.35±1.23	0.73	3.13±0.89	0.74	1.05±1.29	0.37	2.45±1.25	0.72	3.56±0.89	0.52
2	0.37±0.80	0.76	3.77±0.53	0.72	3.04±1.13	0.78	2.98±1.18	0.69	3.24±1.17	0.51
3	0.43±0.95	0.75	3.32±0.96	0.73	0.32±0.85	0.44	3.11±1.10	0.70	2.84±1.37	0.44
4	0.52±0.90	0.73	3.67±0.65	0.75	0.73±1.08	0.39	3.53±0.82	0.73	2.94±1.12	0.45
5	0.35±0.84	0.76	3.52±0.83	0.73	0.26±0.71	0.47	2.53±1.25	0.77	3.38±1.10	0.53
6	0.76±1.04	0.75	3.73±0.84	0.71	0.61±0.97	0.35	3.37±0.92	0.71	2.99±1.14	0.49
7	0.71±1.11	0.71	2.53±1.39	0.76			2.68±1.08	0.72	2.73±1.12	0.48

α*(del)–Chronbach’s alpha if item deleted

### Concurrent validity

Concurrent validity was assessed by Pearson correlation between subscales of FACT-C and EORTC-QLQ-C30 and DASS questionnaires, because stress, depression and anxiety are well-known confounding factors in the assessment of HRQoL. They are presented in Table 4.

**Table 4:** Concurrent validity (Pearson correlation) of the Functional Assessment of Cancer Therapy-Colorectal (FACT-C).

	PWB score	SWB score	EWB score	FWB score	CCS score	FACT-G total score	FACT-C total score	FACT TOI score
EORTC-QLQ-C30								
Functional scales								
QL	0.467*	0.072	0.372*	0.534*	0.415*	0.516*	0.533*	0.565*
PF	0.553*	0.081	0.386*	0.484*	0.368*	0.536*	0.537*	0.561*
RF	0.521*	0.025	0.171	0.375*	0.445*	0.394*	0.441*	0.530*
EF	0.377*	0.149	0.704*	0.520*	0.338*	0.613*	0.592*	0.496*
CF	0.217	0.191	0.226	0.115	0.045	0.256	0.221	0.152
SF	0.383*	0.215	0.356*	0.340*	0.280*	0.452*	0.445*	0.400*
Symptoms scales								
FA	-0.690*	0.029	-0.344*	-0.442*	-0.503*	-0.520*	-0.560*	-0.647*
NV	-0.463*	-0.001	-0.154	-0.219	-0.366*	-0.300*	-0.343*	-0.411*
PA	-0.604*	0.012	-0.204	-0.221	-0.384*	-0.364*	-0.400*	-0.475*
DY	-0.271	-0.171	-0.370*	-0.355*	-0.307*	-0.409*	-0.416*	-0.372*
SL	-0.304*	-0.121	-0.280*	-0.339*	-0.225	-0.369*	-0.362*	-0.348*
AP	-0.485*	0.040	-0.248	-0.406*	-0.519*	-0.398*	-0.464*	-0.556*
CO	-0.095	0.006	-0.012	-0.035	-0.126	-0.049	-0.074	-0.099
DI	-0.296*	-0.277	-0.157	-0.211	-0.332*	-0.308*	-0.341*	-0.329*
FI	-0.154	-0.091	-0.187	-0.120	-0.043	-0.191	-0.168	-0.129
QLQ TOTAL	0.740*	0.152	0.496*	0.567*	0.603*	0.689*	0.724*	0.757*
DAS								
DAS-S	-0.348*	-0.319*	-0.731*	-0.508*	-0.272*	-0.661*	-0.613*	-0.454*
DAS-D	-0.460*	-0.256*	-0.729*	-0.584*	-0.347*	-0.708*	-0.672*	-0.559*
DAS-A	-0.471*	-0.208	-0.664*	-0.468*	-0.324*	-0.632*	-0.604*	-0.506*

Significance differences (p<0.001) between subscales by Pearson chi-squared test

EORTC-QLQ-C30 –European Organization for Research and Treatment of Cancer Colorectal Cancer-Specific Quality of Life Questionnaire: Global health status-QL, Physical functioning-PF, Role Functioning-RF, Emotional functioning-EF, Cognitive functioning-CF, Social functioning SF, Fatigue-FA, Nausea and vomiting-NV, Pain-PA, Dyspnea-DY, Insomnia-SL, Appetite loss-AP, Constipation-CO, Diarrhea-DI, Financial difficulties-FI

It was found that FACT—C subscales correlated with subscales of other instruments. Low correlation was observed between SWB subscales and SF in EORTC-QLQ-C30 scales. EORTIC questionnaire and FACT have opposite direction of scoring. In EORTIC questionnaire, the higher the scores, the better the quality of life, while in FACT, the higher the scores, the higher the symptoms and lower quality of life. It is expected that the direction of correlation is opposite due to the opposite grading of better and worse.

### Reliability

The reproducibility of the questionnaires was studied among 30 patients who answered the questionnaires at the begging and second time after two weeks (Table 5). Table 5 shows the mean, standard deviation of scores collected at baseline and two weeks retest, and reliability of FACT-C subscales and total score. The reproducibility of the FACT-C was almost perfect in all subscales (0.81–0.88), good in PWB scale (0.77) and moderate in EWB subscale (0.57).

**Table 5:** Reliability of FACT-C subscales.

FACT-C subscales	Score		Paired t test p value	ICC^a^
	Test	Retest		
PWB	24.67±3.15	25.07±2.97	0.412	0.773 (0.524–0.892)
SWB	25.15±3.17	24.60±2.72	0.125	0.882 (0.753–0.944)
EWB	20.90±3.22	21.40±2.42	0.387	0.572 (0.102–0.796)
FWB	22.13±4.26	21.97±4.45	0.781	0.838 (0.659–0.923)
CCS	22.42±3.51	22.91±3.23	0.310	0.826 (0.635–0.917)
FACT-G total score	92.85±8.85	93.03±8.99	0.882	0.843 (0.671–0.925)
FACT-C total score	115.27±10.50	115.94±9.90	0.653	0.814 (0.608–0.911)
FACT TOI	69.22±8.81	69.94±7.64	0.555	0.807 (0.595–0.908)

^a^ICC-Interclass correlation coefficient

## Discussion

This was the first psychometric study on the validity and reliability of Serbian version of the FACT-C. Data from the Cancer Registry of the Institute of Public Health of Serbia "Dr Milan Jovanović Batut" from 2018 show that colorectal cancer in Serbia is the second most common cancer in both men and women, while the second leading cause of death from malignant diseases in men and the third in women. According to these data, the number of newly diagnosed cases of colorectal cancer in the 2018 was 4646 (2876 men, 61%) [24]. In our sample percentage of male gender is 54% which is similar to the population gender distribution, but a bit biased toward female gender. The study sample is similar to CRC population in Serbia, with the average age in seventh decade, majority males.

The proportion of missing data was relatively low, and that was mostly for FACT-C, GS scale, satisfaction with sexual life item (GS7) which proved to be similar with the results of other studies, like French and Chinese validation study [17, 19].

Internal consistency of FACT—C questionnaire was good for all subscales except for Colorectal Cancer Subscale (CCS) where the Cronbach’s alpha was 0.53. It was similar to the results of other studies, like Chinese and Korean, where Cronbach’s alpha for CCS scale was lowest among all scales, where it was 0.62, or 0.68 [18, 19].

Reliability of FACT subscales is satisfactory for subscales PWB, SWB and FWB subscales. These subscales have low change of alpha if item deleted compared to all items together. Subscales EWB and CCS have lower Cronbach’s alpha level compared to other three. Even though CCS has lower alpha in total, using alpha change if item deleted method reveals no significant change in alpha. This means that removing any of items from this subscale will not improve consistency of the subscale. EWB subscale reveals high variation in alpha if item deleted, especially item 1, 4 and 6. Actually, if item 2 is deleted the alpha level increases, while deletion of other two makes significant decrease of alpha level in this subscale.

These validity issues in this questionnaire are mostly due to scaling direction of the specific questions. In EWB subscale, one question “I am satisfied with how I am coping with my illness” has high influence of Cronbach’s alpha. When this question is deleted, alpha reaches reasonable, and similar to other domains, 0.78 value. The problem with this question is that it is the question with the opposite scale direction (the higher the worse) compared to the other question. It is very likely that this question was confusing and the patients linked the higher score with worse status. That might be the reason why this question was inconsistent with the others in this domain and its’ deletion increases the consistency of the domain.

In CCS subscale, first two questions have the-higher-the-worse scoring direction while 3^rd^ and 4^th^ have the opposite. Then, 5^th^ question again has the-higher-the-worse and then, again, the last two questions (6^th^ and 7^th^) have the opposite scoring direction. It is very likely that this direction changes in this domain are confusing, and the patients, who are 68 years in average, are confused and hard to follow the scoring direction. The author of the questionnaire might consider the next version of questionnaire with the grading system in same direction.

Exploring the floor and ceiling effect reveals acceptable percentage of participants with floor effect. However, ceiling effect of PWB, SWB and EBW is higher than standard used percentage of 15%, which might suggest inability of questionnaire to distinguish among those at high end of the spectrum regarding these three domains.

Concurrent validity was examined using EORTC-QLQ-C30 questionnaire. This questionnaire consist of two sets of subscales, functional scales and symptoms scales. Using this questionnaire as already validated comparator, the correlation between totals and subscales with FACT-C revealed high level of agreement. Total scores of questionnaires have high correlation coefficients, between 0.689–0.757. FACT-C subscales, also, have satisfactory level of agreement with EORTC-QLQ-C30 functional and symptoms subscales. The only sucbscale with lower correlation is SWB subscore, without significant correlation with Social functioning domain of EORTC-QLQ-C30 questionnaire. Since CCS score consists of symptoms and specific questions regarding colorectal cancer, it is expected to have significant correlation with symptoms scale as well as with functional scales of EORTIC-QLQ-C30.

Our study confirmed test-retest reliability, resulting in substantial reproducibility (ICC>0.7) for all of subscales except for EWB subscale (ICC = 0.57). But, reviewing the literature findings, we found similar results compared to other authors (versions), like Chinese or French [17, 19].

## Conclusions

The study showed that the Serbian version of the FACT—C questionnaire is an appropriate instrument tool for examining quality of life in patients with colorectal cancer. It is supported by its correlations with other validated scales, such as EORTC-QLQ-C30 and DASS. It has satisfactory psychometric properties for the usage in Serbian population and we recommend its application in clinical practice.

## Limitation

A limitation of our study was that interviewed patients were from single hospital in Belgrade, however, this hospital is one of the leading centers and patients were of different socio-economic status and at different stages of the disease. The highest percentage of patients had classic surgery (55%), but there were also patients who were not hospitalized. The percentage of patients with complications was very small.

## Supporting information

S1 File English version of FACT-C questionnaire. (PDF)

S2 File Serbian version of FACT-C questionnaire. (PDF)

S1 Data Database of FACT-C validation. (XLSX)
