# Supplementary material for: Transcultural adaptation and validation of the Serbian version of the colorectal-specific quality of life questionnaire FACT-C
Source: PLoS One. 2022 Feb 3;17(2):e0263110. doi: 10.1371/journal.pone.0263110 (PMC8812893; doi:10.1371/journal.pone.0263110)
Supplement: S1 File — (PDF) [file pone.0263110.s001.pdf]

FACT-C (Version 4)

Below is a list of statements that other people with your illness have said are important. **Please circle or mark one number per line to indicate your response as it applies to the <u>past 7 days</u>.**

	**PHYSICAL WELL-BEING**	**Not at all**	**A little bit**	**Some-what**	**Quite a bit**	**Very much**
GP1	I have a lack of energy	0	1	2	3	4
GP2	I have nausea	0	1	2	3	4
GP3	Because of my physical condition, I have trouble meeting the needs of my family	0	1	2	3	4
GP4	I have pain	0	1	2	3	4
GP5	I am bothered by side effects of treatment	0	1	2	3	4
GP6	I feel ill	0	1	2	3	4
GP7	I am forced to spend time in bed	0	1	2	3	4

	**SOCIAL/FAMILY WELL-BEING**	**Not at all**	**A little bit**	**Some-what**	**Quite a bit**	**Very much**
GS1	I feel close to my friends	0	1	2	3	4
GS2	I get emotional support from my family	0	1	2	3	4
GS3	I get support from my friends	0	1	2	3	4
GS4	My family has accepted my illness	0	1	2	3	4
GS5	I am satisfied with family communication about my illness	0	1	2	3	4
GS6	I feel close to my partner (or the person who is my main support)	0	1	2	3	4
Q1	*Regardless of your current level of sexual activity, please answer the following question. If you prefer not to answer it, please mark this box* ☐ *and go to the next section.*					
GS7	I am satisfied with my sex life	0	1	2	3	4

Copyright 1987, 1997

FACT-C (Version 4)

Please circle or mark one number per line to indicate your response as it applies to the <u>past 7 days</u>.

	**<u>EMOTIONAL WELL-BEING</u>**	**Not at all**	**A little bit**	**Some-what**	**Quite a bit**	**Very much**
GE1	I feel sad	0	1	2	3	4
GE2	I am satisfied with how I am coping with my illness	0	1	2	3	4
GE3	I am losing hope in the fight against my illness	0	1	2	3	4
GE4	I feel nervous	0	1	2	3	4
GE5	I worry about dying	0	1	2	3	4
GE6	I worry that my condition will get worse	0	1	2	3	4

	**<u>FUNCTIONAL WELL-BEING</u>**	**Not at all**	**A little bit**	**Some-what**	**Quite a bit**	**Very much**
GF1	I am able to work (include work at home)	0	1	2	3	4
GF2	My work (include work at home) is fulfilling	0	1	2	3	4
GF3	I am able to enjoy life	0	1	2	3	4
GF4	I have accepted my illness	0	1	2	3	4
GF5	I am sleeping well	0	1	2	3	4
GF6	I am enjoying the things I usually do for fun	0	1	2	3	4
GF7	I am content with the quality of my life right now	0	1	2	3	4

Copyright 1987, 1997

FACT-C (Version 4)

Please circle or mark one number per line to indicate your response as it applies to the past 7 days.

	ADDITIONAL CONCERNS	Not at all	A little bit	Some-what	Quite a bit	Very much
C1	I have swelling or cramps in my stomach area	0	1	2	3	4
C2	I am losing weight	0	1	2	3	4
C3	I have control of my bowels	0	1	2	3	4
C4	I can digest my food well	0	1	2	3	4
C5	I have diarrhea (diarrhoea)	0	1	2	3	4
C6	I have a good appetite	0	1	2	3	4
C7	I like the appearance of my body	0	1	2	3	4
Q2	Do you have an ostomy appliance? (Mark one box)	☐ No		or	☐ Yes	
	If yes, please answer the next two items:					
C8	I am embarrassed by my ostomy appliance	0	1	2	3	4
C9	Caring for my ostomy appliance is difficult	0	1	2	3	4

Copyright 1987, 1997
