# Supplementary material for: Transcultural adaptation and validation of the Serbian version of the colorectal-specific quality of life questionnaire FACT-C
Source: PLoS One. 2022 Feb 3;17(2):e0263110. doi: 10.1371/journal.pone.0263110 (PMC8812893; doi:10.1371/journal.pone.0263110)
Supplement: S2 File — (PDF) [file pone.0263110.s002.pdf]

FACT-C (Version 4)

Ispod je lista izjava za koje su osobe sa Vašom bolešću rekle da su važne. **Molimo Vas da zaokružite ili obeležite jedan broj u svakom redu da biste označili odgovor koji se odnosi na <u>poslednjih 7 dana</u>.**

	FIZIČKO STANJE	Nimalo	Malo	Donekle	Dosta	Veoma mnogo
GP1	Nedostaje mi energija	0	1	2	3	4
GP2	Imam mučninu	0	1	2	3	4
GP3	Zbog mog fizičkog stanja imam problema u zadovoljavanju potreba moje porodice	0	1	2	3	4
GP4	Imam bolove	0	1	2	3	4
GP5	Muče me propratne pojave lečenja	0	1	2	3	4
GP6	Osećam se bolesno	0	1	2	3	4
GP7	Primoran/a sam da vreme provodim u postelji	0	1	2	3	4

	DRUŠTVENO/PORODIČNO OKRUŽENJE	Nimalo	Malo	Donekle	Dosta	Veoma mnogo
GS1	Blizak/ska sam sa mojim prijateljima	0	1	2	3	4
GS2	Dobijam emocionalnu podršku od moje porodice	0	1	2	3	4
GS3	Dobijam podršku od mojih prijatelja	0	1	2	3	4
GS4	Moja porodica je prihvatila moju bolest	0	1	2	3	4
GS5	Zadovoljan/a sam kako se u mojoj porodici razgovara o mojoj bolesti	0	1	2	3	4
GS6	Osećam se blisko sa mojim partnerom (ili osobom koja je moja glavna podrška)	0	1	2	3	4
Q1	*Bez obzira na sadašnji nivo Vaše seksualne aktivnosti molimo da odgovorite na sledeće pitanje. Ako ne želite da odgovorite molimo Vas da stavite znak u ovaj kvadrat i pređete na sledeći deo.* ☐					
GS7	Zadovoljan/a sam mojim seksualnim životom	0	1	2	3	4

Copyright 1987, 1997

FACT-C (Version 4)

Molimo Vas da zaokružite ili obeležite jedan broj u svakom redu da biste označili odgovor koji se odnosi na <u>poslednjih 7 dana</u>.

	**EMOCIONALNO STANJE**	**Nimalo**	**Malo**	**Donekle**	**Dosta**	**Veoma mnogo**
GE1	Osećam se tužno	0	1	2	3	4
GE2	Zadovoljan/a sam kako se nosim sa svojom bolešću	0	1	2	3	4
GE3	Gubim nadu u borbi protiv moje bolesti	0	1	2	3	4
GE4	Osećam se nervozno	0	1	2	3	4
GE5	Brine me smrt..	0	1	2	3	4
GE6	Brinem da će se moje stanje pogoršati...................	0	1	2	3	4

	**FUNKCIONALNO STANJE**	**Nimalo**	**Malo**	**Donekle**	**Dosta**	**Veoma mnogo**
GF1	Sposoban/a sam da radim (uključujući i rad kod kuće)...	0	1	2	3	4
GF2	Moj posao (uključujući i onaj u kući) me ispunjava	0	1	2	3	4
GF3	Sposoban/a sam da uživam u životu	0	1	2	3	4
GF4	Prihvatio/la sam moju bolest.................................	0	1	2	3	4
GF5	Dobro spavam	0	1	2	3	4
GF6	Uživam u stvarima koje obično činim za zabavu ...	0	1	2	3	4
GF7	Zadovoljan/a sam kvalitetom mog sadašnjeg života..	0	1	2	3	4

Copyright 1987, 1997

FACT-C (Version 4)

Molimo Vas da zaokružite ili obeležite jedan broj u svakom redu da biste označili odgovor koji se odnosi na <u>poslednjih 7 dana</u>.

	**DODATNE BRIGE**	**Nimalo**	**Malo**	**Donekle**	**Dosta**	**Veoma mnogo**
C1	Imam otekline ili grčeve u predelu stomaka	0	1	2	3	4
C2	Gubim na težini	0	1	2	3	4
C3	Imam kontrolu nad stolicom	0	1	2	3	4
C4	Mogu dobro da svarim hranu	0	1	2	3	4
C5	Imam proliv	0	1	2	3	4
C6	Imam dobar apetit..	0	1	2	3	4
C7	Volim izgled moga tela	0	1	2	3	4
Q2	Da li imate stomu? (Stavite znak u jedan kvadrat)	☐ Ne		ili	☐ Da	
	Ako da, odgovorite na sledeća dva pitanja:					
C8	Stoma mi pričinjava neugodnost......................................	0	1	2	3	4
C9	Održavanje moje stome je teško.......................................	0	1	2	3	4

Copyright 1987, 1997
